# Kennicutt-Schmidt Law in the Central Region of NGC 4321 as Seen by ALMA

**DOI:** 10.1038/srep26896

**Published:** 2016-06-01

**Authors:** Jazeel H. Azeez, C.-Y. Hwang, Zamri Z. Abidin, Zainol A. Ibrahim

**Affiliations:** 1 University of Malaya, department of Physics, Kuala Lumpur, 50603, Malaysia; 2 AL-Nahrain University, department of Physics, Baghdad, 10072, Iraq; 3 National Central University, Graduate Institute of Astronomy, Chung-Li, 32054, Taiwan

## Abstract

We present the Atacama Large Millimeter/Sub-millimeter Array (ALMA) cycle-0 science verification data of the CO(1–0) line emission in the central region of NGC 4321 (also known as M100) at the distance of 17.1 Mpc and VLA, L-band data of HI of the same galaxy. We have drawn the center area of M100 in the ^12^CO(J = 1–0) line with the resolution of (3.87″ × 2.53″) as viewed by ALMA, along with HI and Spitzer 8 and 3.6 μm data. The relationship between the surface density of molecular gas mass ∑H_2_ and that of star formation rate ∑SFR has been investigated, in addition to the relationship between the surface density of the neutral atomic hydrogen mass and that of ∑SFR (Kennicutt–Schmidt law) in this galaxy with a high spatial resolution. The results indicate that a significant correlation exists between the SFR surface density and the molecular gas mass density in the ~2 kpc region. The power-law index has been determined for three regions: center, upper and lower arms. The value of this index in the center region is 1.13, which follows the traditional (K-S) law and indicates that the molecular gas is affected by star formation.

One of the most crucial processes that affect the evolution of galaxies is star formation, particularly of massive ones. This condition makes this branch of research an active field in modern astronomy. One of the key points in studying the star formation physics of massive stars is understanding the molecular gas dynamics and their interactions^1^. Based on observations of galaxies, a correlation generally exists between the surface density of star formation (SFR) and the molecular gas, which can be expressed as ∑SFR_(∑gas)^n^, where ∑SFR represents the SFR per unit area and ∑gas represents the surface density of gas. This relation is called the Kennicutt–Schmidt (K-S) law^2^,^3^.

The molecular gas ∑H_2_ is found to have a better correlation with the average surface density of SFR at the disk than the total (HI + H_2_) gas^4^. The physics behind this correlation remains unclear today. In the star formation law; the exponent *n* is primarily distinguishing the dominant mechanism that controls the formation of stars, which in turn provides a certain form of the K-S law^5^. K-S law is derived on sub-galactic scales for nearby galaxies^6^,^7^. Lately, Kennicutt^8^ examined the K-S law in M51 within the range of 500 pc with a cloud mass of 10^6^ M_⊙_–10^7^ M_⊙_. Verly^9^ demonstrated the existence of a weak correlation at the 180 scale despite the strong correlation at the global scale, as has been shown by Heyer^10^. They claimed that the non-linear K-S law is applicable down to that scale. Bigiel^6^ disclosed that the K-S law is applied in seven galaxies at 750 pc scale, and it is correlated down to the 250 pc scale for M51.

NGC 4321 (also known as M100) is an SAB(s) bc galaxy located in the Virgo cluster. This galaxy has two symmetric well-defined spiral arm^11^. The relative propinquity of this galaxy is (17.1 Mpc)^12^, and studying the central distribution and kinematics of the HI and H_2_ components is possible because of the mild inclination angle of the galaxy^13^. Cepa^13^ studied numerous locations in the interarm and southern spiral arm of NGC 4321. The telescope used was NRO-45 m. This galaxy is a grand design galaxy.

Studying dust distribution is important in understanding the evolution and formation of stars in this, and any other, galaxy. The accretion rate of gases and their conversion into stars control not only the formation history of stars within galaxies, but also their chemical development^14^. Sheth^15^ used the BIMA survey of a nearby galaxy to compare the star formation with the molecular gas distribution. Identifying the HI distribution within the galaxy is important in understanding the role of atomic hydrogen during the star formation process. A comparison is made with H_2_ (from CO) along the inner and outer regions of the arms to determine the prominent phase of neutral gas and the variations in the ratio of molecular to atomic gas^16^. The interstellar medium and gas reservoirs can be studied based on CO molecular observations^17^. The HI 21-cm hyperfine transition provides insight into the atomic gas (or neutral hydrogen)^18^, and the carbon monoxide, mostly ^12^CO(J = 1–0), provides trace to the molecular gas (typically H_2_)^19^,^20^,^21^. Recently, considerable efforts have been exerted to study the correlation between tracing gases and star formation, particularly in nearby galaxies^22^,^23^,^24^,^25^. The CO rotational transitions are used for tracing the molecular gas to explore the star formation rates (SFRs), the molecular gas properties and the dynamics of galaxies^26^. The bulk of the galactic structure is traced by observing 21-cm HI transition by observing delineates neutral atomic gas more precisely^27^.

We use the ALMA SV data of the CO(1–0) emission line for galaxy NGC 4321 with z = 0.005 to investigate the molecular gas ∑H_2_ within their clumps. More precisely, our goal is to determine the SFR and gas mass surface densities on sub-galactic scales and examine their correlation. This paper is structured as follows. The data for molecular and atomic gases are described in Section 2. The distribution of CO(1–0) and HI is presented in Section 3.1. The mass of neutral atomic and molecular gases is discussed in Section 3.2. The relationship between the SFR surface density and gas mass density is mentioned in Section 3.3. The summary is provided in section 4.

## Data

### CO(J = 1–0)

NGC 4321 has been observed using ALMA (Atacama Large Millimeter = Submillimeter Array) in the cycle-0 science verification data with band 3 (λ ~ 2.6 mm) receivers. There are three sets of observations of ALMA data; The first set is the data observed for NGC 4321 with 12-m array on August 10 and September 10, 2011. The observation formed of 47 pointing mosaics centered at RA = 12:22:54.6, Dec = +15:48:56.5 with four spectral windows. The second one, the data taken for NGC 4321 with 7-m array on March 17–18, April 14 and May 11, 2013. The observations consisted from 23 pointing mosaics centered at RA = 12:22:54.3, Dec = +15:48:51.4 with either two or four central windows. The third one is the single dish data for NGC 4321, which was taken on July 1, 5, 7 and 17, 2014, with a total power array.

The restoring beam of the CO feather image for M100 is 3.87″ × 2.53″. The systemic velocity employed was 1575 km s^−1^, which was obtained from(Knapen *et al.*^16^) and the distance of 17.1 Mpc taken from (Yuan and Kuo)^12^. At 17.1 Mpc, the angular-to-linear scale is ~82 pc**/**″. The analysis was conducted using the Common Astronomy Software Application (CASA).

### HI

The HI data of spiral galaxy NGC 4321 were obtained from the Very Large Array (VLA) data archive with project number AS0750^28^. The source was observed on March 25, 2003, for 12,900 s. The observation was single pointing performed in the D-configuration (1 km, 44″ angular resolution) of the VLA. The total bandwidth of the observation was 1538.1 kHz centered at a velocity of 1446.2 km s^−1^ and divided in to 64 channels of 24.41 kHz each. The total observational parameters are fully detailed in Table 1. The data were calibrated and reduced in the CASA 4.1 data processing package. These data were combined, and a map with a resolution of 52.98″ × 47.5″ was produced using the Briggs weighting. The visibility data were cleaned in CASA using the task CLEAN. The pixel size was set to CELL = 15″, which was proximately about one-third of the beam size and IMSIZE = 64. Thus, the image contained an area of ~0.09 deg^2^. A clean cube was also produced by first performing a continuum subtraction using the task UVCONTSUB, which removed the continuum emission from the u,v data of all channels, then by applying primary beam correction. A channel map was used as input for the CASA task IMMOMENTS to calculate the total HI (zeroth moment) map.

## Results and Discussion

### Line Emission Distribution

#### CO(1–0)

**Figure 1:**
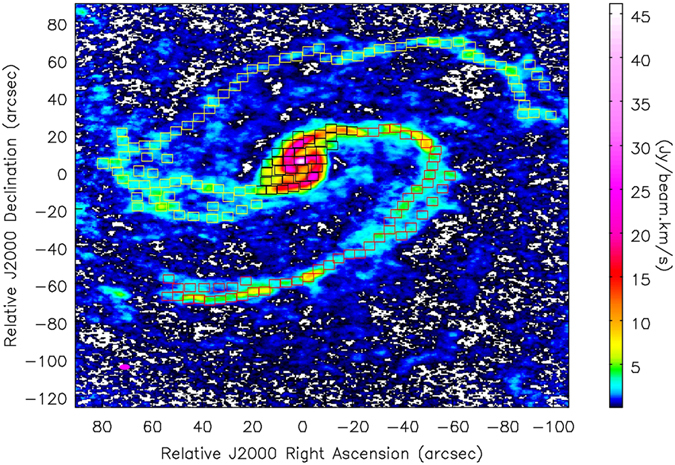
Integrated intensity map of CO(1–0) of the central region of NGC 4321. The intensity map is divided into boxes distributed on three main regions: the nucleus (black boxes), lower arm (red boxes) and upper arm (yellow boxes). The size of each box is 4″ × 4″, which corresponds to 331.6 pc × 331.6 pc.

Figure 1 shows the CO(1–0) integrated intensity map. Where the CO emission trace clearly the two arms that are highly contrasted with spiral pattern. The two arms emerge from the end of a huge gaseous bar, which corresponds to the stellar bar observed in the infrared images of the galaxy. Arm 1 (lower arm) emerges from the east part and gradually extends towards the south, which is stronger on average, and it can be tracked more continuously than arm 2 (upper arm). The later emerges from the west and it splits up into two armlets that meet at approximately *r* = 6″, then disappear and finally show up in the north.

#### HI

**Figure 2:**
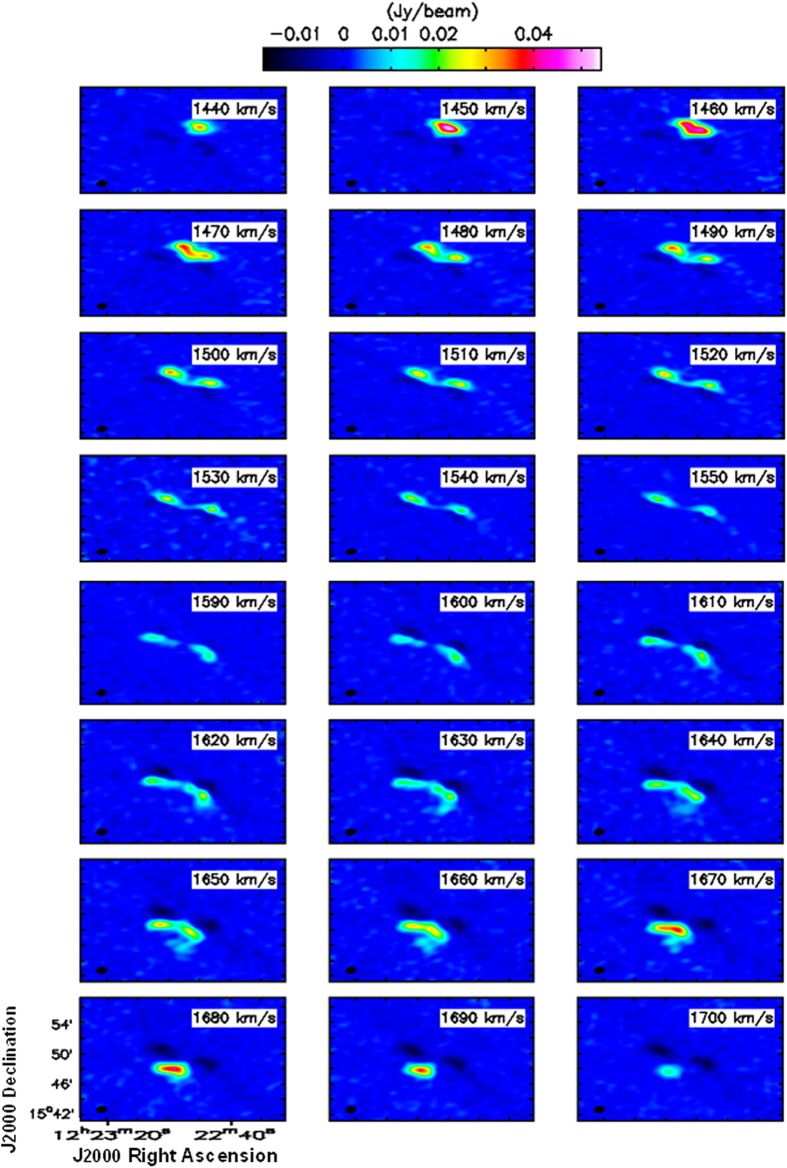
Velocity-channel map of the HI emission in the central region of NGC 4321 as seen by VLA.

Figure 2 shows the velocity-channel map of the neutral atomic hydrogen gas (HI) in the central region of the spiral galaxy NGC 4321 at the resolution of 52.98″ × 47.5″. The HI emission is detected in 27 of the channel maps of the 10 km s^−1^ velocity width. The rms noise in the channel map is 0.99 mJy beam^−1^, and the beak emission is 58.8 mJy beam^−1^ in the channel 1450 km s^−1^. The total HI flux detected in the beam is 47.3 Jy km s^−1^. No HI bridge is visible in the data either in the channel maps or in the intensity map.

**Figure 3:**
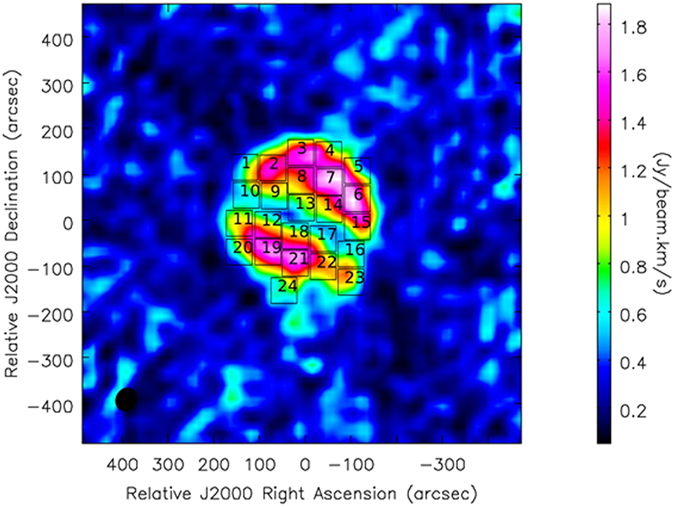
Integrated intensity map of HI of the central region of NGC 4321. We also plot 24 boxes that are used to calculate the neutral atomic hydrogen surface density and mass.

Figure 3 shows the intensity map of the HI gas (zeroth map), which is derived from the Briggs weighted channel map without Hanning smoothing. A relative deficiency of HI is observed in the center, although the gas is detected over almost the entire disk. The HI gas distribution is extended toward the upper and lower arms. The disk of NGC 4321 is symmetrical. The spiral arm could not be detected because of poor resolution of these data. Based on the CO(1–0) data of the same galaxy that show a peak in the center region and decline in the disk, it is clear that the lack in the center of the galaxy does not imply a lack of gas there.

### Gas Mass and Surface Density

#### Molecular Gas CO(1–0)

The molecular gas bulk is traced by CO(1–0)^29^. Accordingly, the CO(1–0) integrated intensity map can be used to calculate the molecular gas mass and surface density. The mass of the molecular gas in the integrated intensity map can be estimated by the CO flux using the CO-to-H_2_ conversion factor. If the conversion factor is assumed to be X = 2.3 × 10^20^ cm^−2^ (K km s^−1^)^−1^ (see Burillo *et al.*^30^), the CO flux can be converted into the mass of the molecular hydrogen using the following formula (assuming the distance D = 17.1 Mpc):


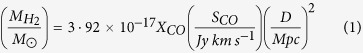


The total molecular gas mass *M*(*H*_2_) within the total area *∆x* × *∆y* = 200″ × 200″ (as shown in Fig. 1) is equal to 0.9 × 10^10^ M_⊙_ at D = 17.1 Mpc. The molecular gas mass in the center region at *r* = 30″ is equal to 3.1 × 10^9^ M_⊙_, which is close to the result of *M*(*H*_2_) = 2.8 × 10^9^ M_⊙_ at *r* = 30″ from the IRAM single dish observation^31^ and similar to the result of *M*(*H*_2_) = 3.1 × 10^9^ M_⊙_ from the JCMT^32^. This result indicates that no missing flux exists in the central region at *r* = 30″. A direct measurement is unavailable for the conversion factor in M100. Thus, an error of the mass of the molecular gas estimated using the galactic conversion factor is possible. The central 14.5 kpc region of the NGC 4321 galaxy is divided into 174 regions covering the nucleus (black boxes), lower arm (red boxes) and upper arm (yellow boxes), as shown in Fig. 1. The size of each box is 4″ × 4″, which corresponds to 331.6 pc × 331.6 pc. The molecular hydrogen mass and density for each region are obtained and listed in Table 2. The surface density values are in terms of hydrogen alone, i.e. the contribution of Helium hasn’t been included. A strong concentration of molecular gas mass is observed toward the nucleus (black boxes region) that contains ~20.5% of the total H_2_ content in the sample region *∆x* × *∆y* = 200″ × 200″.

### Neutral Atomic Gas HI

The intensity map is divided into 61 regions (see Fig. 3), and each region is 55″ × 55″ (4560 pc × 4560 pc). The values of the HI surface density, HI mass, SFR and SFR surface density are derived for each region, and the results are summarized in Table 3. The total mass of the atomic hydrogen gas can then be calculated, using the following equation^16^:


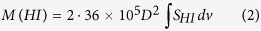


where *M*(*HI*) is expressed in solar mass. The distance *D* in Mpc and the flux integral are in *Jy km s*^−1^. The total *M*(*HI*) = 3.02 × 10^9^ M_⊙_ is found at the assumed distance of 17.1 Mpc. The values of this quantity as published in the literature vary. Kenapen^16^ provides a value of 2.6 × 10^9^ M_⊙_, at the assumed distance of 13.8 Mpc. Lewis^33^ presents a value of 2.3 × 10^9^ M_⊙_, corrected for the distance of 13.8 Mpc. Huchtmeier^34^ provides a value of *M*(*HI*) = 3.3 × 10^9^ M_⊙_ (D = 13.8 Mpc). All of the above mentioned studies were made with various telescopes.

### Relationship between Gas Mass Density and Star Formation

**Figure 4:**
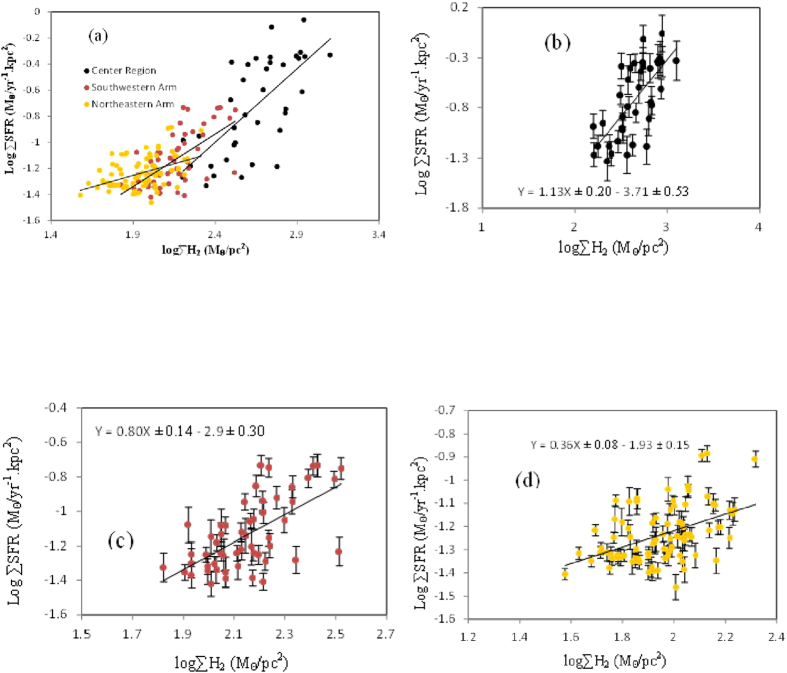
Comparison between the molecular gas mass density (as converted from CO flux from ALMA) and star formation rate surface density of (**a**) Combine all regions together, (**b**) center, (**c**) upper arm and (**d**) lower arm regions. The data points crosses with the error bar in the nucleus (black filled dots), lower arm (red filled dots) and upper arm (yellow filled dots) respectively. The solid line in each figure is the line from the least square fit. The scatter ϵ values for the center, upper and lower regions are 0.27, 0.11 and 0.16 respectively.

The surface density of the molecular gas and SFR is calculated in the nuclear disk region and along the upper and lower arms and then compared with neutral atomic gas. The infrared data are used as star formation tracers. The infrared data are obtained from the Spitzer Space Telescope Infrared Array Camera (IRAC) 3.6 and 8 μm images (http://ssc.spitzer.caltech.edu/archanaly/archive.html). These data are background-subtracted images. Wu^35^ showed that the dust emission at 8 μm can be used as a star formation indicator. The dust emission in the central and spiral arms of the NGC 4321 is estimated using the observed Spitzer IRAC 8 and 3.6 μm fluxes applying the following equation^29^:


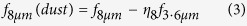


where *η*_8_ = 0.232^36^. To enhance the visibility of the dust features, the contribution from starlight (measured at 3.6 μm) has been subtracted from the 8 μm image. The derived values of the molecular and neutral atomic gases are shown in Tables 2 and 3. The star formation surface density (∑SFR) is derived from the 8 μm dust luminosity^29^


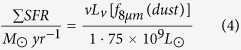


where L*v* is the 8 μm dust luminosity. The derived values are shown in Tables 2 and 3. The relationship between the SFR and molecular gas surface densities is drawn for NGC 4321 with the spatial resolution of 4″ × 4″ as shown in Fig. 4a. The data are colorized by region, namely, center region (black circle), upper arm (yellow circle) and lower arm (red circle) to distinguish these regions. Numerous differences are observed between the SFR surface density and the distribution of the molecular gas mass density in each region. For each region ordinary least square fitting was used. The uncertainties for the slope and the intercept for each region are calculated using python, they refer to the fitting error. For the center region (black box), the derived power-law index of ∑gas − ∑SFR relation is equal to 1.13 ± 0.20 with intercept −3.71 ± 0.53. This value is within the range of the traditional K-S law, which is around 1–2.0. This linear relation is interpreted as star formation efficiency (SFE, the SFR divided by M(H_2_)) is constant. This result can be interpreted as an indication that external processes or feedback mechanisms that control the gas supply are important for regulating star formation in massive galaxies^37^. The constant star formation efficiency (SFE) has been found in many spiral galaxies as shown in^38^. In addition to that Wilson^32^ has found a uniform depletion time (SFE)^−1^ for the central region of M100 spiral galaxy. However they found that the CO J = 3–2 line may represent more reliable tracer of the dense molecular gas than is the CO J = 1–0. For the lower arm (red boxes), the index of K-S is 0.8 ± 0.14 with intercept −2.86 ± 0.3. Although this value is smaller than the traditional K-S law. It’s common for K-S law to relate the observed CO luminosity to the number of star forming clouds, assuming these clouds to have isotropic properties such as volume density, SFR and efficiency of cloud. However for the observations at resolution >100 pc, instead of resolving the individual clouds, the CO flux is rather dispersed throughout the beam. In such case, regions with abundant clouds will emit more CO directly proportional to the number of these clouds. These conclusions are clearly supported by the studies^39^,^40^,^41^,^42^ specially for the N values ~0.6–0.8, which are close to our results. The sub-linear K-S relationship indicates that the clouds have non-isotropic properties for the SFR and the surface density may vary through different regions in the galaxy as its clear form our results in different regions^43^. For the upper arm (yellow boxes), the power-law index of the K-S law relation is approximately 0.36 ± 0.08 with intercept 1.93 ± 0.15. This value is significantly lower than a typical power law index of the K-S law. One possible explanation of this result is that the gas may be under a sub-thermal condition, which would produce a flatter K-S law, as shown by Narayanan^44^. CO(1–0) is a fundamental unit of star formation, but the non-linear ∑gas − ∑SFR correlation in the lower and upper arms demonstrates that these regions do not form stars at a fixed rate per unit mass^44^. These observations suggest that molecular cores may represent a fundamental unit of star formation. Moreover, a significant correlation exists between SFR surface density and molecular gas mass surface density in the ~2 kpc region (black boxes). The correlation coefficient *r* = 0.69 with 99.9% probability.

**Figure 5:**
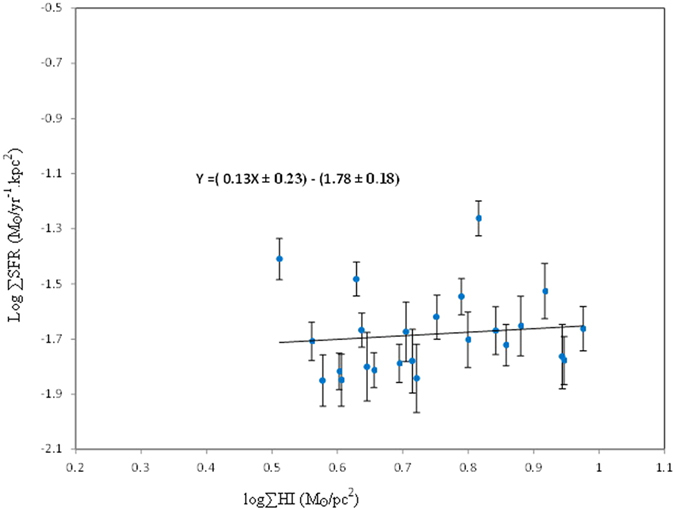
Relation between the surface density of the neutral atomic hydrogen ∑HI and surface density of star formation rate ∑SFR. The data points crosses with the error bars. The solid line represents the linear square fit line.

**Figure 6:**
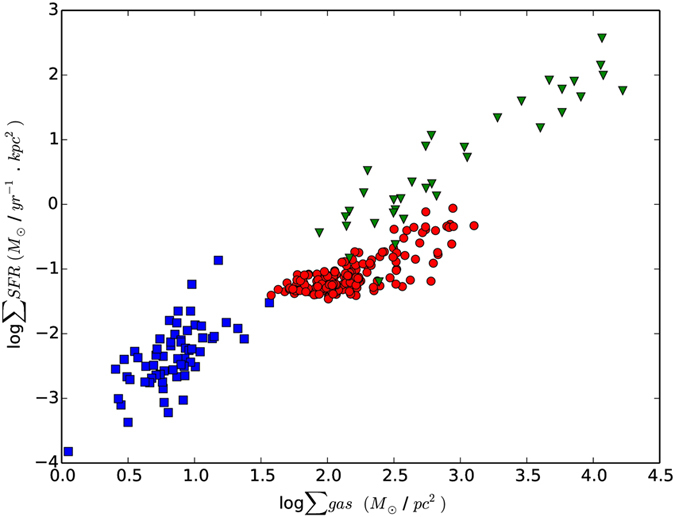
Relation between the surface density of molecular gas and ∑SFR for normal galaxies (blue squares), starburst galaxies (green triangle), and NGC 4321 (red circles). The gas surface density is derived from CO(1–0) emission. The normal and star burst galaxy samples are obtained from.

To study the K-S law behavior at different regions as in Fig. 5, which shows the relationship between the neutral atomic hydrogen and SFR surface densities. The physical interpretation of the SFR versus the HI K-S law is not obvious. However, HI may trace the physical influences of atomic gas density on the SFR, or the density of HI could be regulated through the SFR, by the dissociation of molecular gas by a hot star^2^,^45^,^46^. The relationship between the surface ∑gas and ∑SFR is shown in Fig. 6. The star formation rate surface density of the center and spiral arm regions of NGC 4321 are similar to those of starburst galaxies and considerably higher than those of normal galaxies^2^,^47^. However, they all appear to follow the same star formation law^2^. This result supports the idea that the spiral arms and the center in the inner region are suffering from a starburst.

### Summary and Conclusion

We have drawn the galaxy M100 in ^12^CO(J = 1–0) line with ALMA and compared the emission with the HI 21-cm line from VLA. We have tested the relationship between the surface density of molecular gas and the SFR in an external galaxy with high resolution for three regions, namely, center, upper arm and lower arm. We have concluded that the center region is composed of relatively high density gases that have sufficient intensity to undergo the star formation process. By contrast, the emerged gases for the lower arm region have higher intensity (energy) and more density than the upper arm. Hence, the power law index in the lower arm region is higher than that in upper arm, but still lower than that in the traditional K-S law. The value of the power-law index in the upper arm is significantly low, which is based on the distribution of gases as they emerge from the east bar of the nucleus region and split into two armlets. This split may induce dissipation in the gas energy and reduce the collisions between these gases, which may result in the gas being under the sub-thermal condition. This condition can be observed by the disappearance of gas emission slightly after the two armlets meet in the north.

## Additional Information

**How to cite this article**: Azeez, J. H. *et al.* Kennicutt-Schmidt Law in the Central Region of NGC 4321 as Seen by ALMA. *Sci. Rep.* **6**, 26896; doi: 10.1038/srep26896 (2016).

## Figures and Tables

**Table 1:** VLA Observational Parameters.

Parameter	Value
Target	NGC 4321
Observing date	2003 march 25
Total observation time	12900 s
Field center:	
R.A.(J_2000_)	12^h^ 22^m^ 54.89^s^
Dec.(J_2000_)	+15°49′20.70″
Number of antenna	27
System velocity	1446.2 km s^−1^
Rest frequency	1420.41 MHz
Restoring beam	(major, minor, P.A.)
	(52.98″, 47.5″, −26.3°)
Total bandwidth	1538.1 kHz
Total channels	64
Velocity resolution	10 km s^−1^
Configuration	D
Primary calibrator	1331 + 305
Secondary calibrator	1221 + 282

**Table 2:** Integrated Flux Density, Molecular Gas Density and Mass, *f*_8_ μm (dust) and Star Formation Rate Surface Density.

Area	S_CO_ (Jy km s^−1^)	M(H_2_) × 10^6^ (M_ʘ_)	∑H_2_ (M_ʘ_ pc^−2^)	f_8 _μm(dust) (mJy)	∑SFR × 10^−2^ (M_ʘ_ yr^−1^ kpc^−2^)
1	52.93	139.35	1267.2	23.67	46.78
2	22.89	60.26	547.98	20.64	40.79
3	36.70	96.61	878.51	23.41	46.27
4	35.03	92.22	838.59	24.81	49.04
5	33.05	87.00	791.17	23.17	45.79
6	20.67	54.41	494.75	12.79	25.28
7	13.85	36.45	331.48	5.02	9.91
8	15.65	41.21	374.71	15.17	29.99
9	16.52	43.48	395.38	19.97	39.47
10	8.33	21.93	199.41	5.65	11.17
11	10.36	27.27	248.00	2.78	5.50
12	9.92	26.10	237.38	3.31	6.54
13	7.44	19.57	177.99	3.31	6.54
14	15.98	42.08	382.65	8.19	16.20
15	13.72	36.11	328.40	6.53	12.91
16	35.95	94.66	860.77	12.32	24.36
17	28.38	74.72	679.43	8.47	16.75
18	21.67	57.05	518.76	18.54	36.64
19	22.83	60.11	546.65	22.69	44.85
20	34.30	90.31	821.26	22.24	43.97
21	18.67	49.15	446.94	22.31	44.10
22	13.11	34.51	313.83	10.71	21.17
23	19.19	50.51	459.30	7.18	14.19
24	13.75	36.20	329.15	4.81	9.52
25	17.55	46.21	420.19	3.41	6.74
26	12.35	32.51	295.66	3.70	7.32
27	6.70	17.64	160.42	2.72	5.38
28	6.64	17.49	159.04	5.24	10.35
29	13.24	34.86	317.03	20.85	41.22
30	22.95	60.42	549.41	20.54	40.60
31	27.15	71.47	649.90	19.88	39.30
32	37.05	97.54	886.96	23.01	45.48
33	28.17	74.17	674.51	9.10	18.01
34	26.13	68.79	625.58	6.23	12.31
35	25.11	66.12	601.25	3.29	6.49
36	15.30	40.27	366.17	2.71	5.36
37	9.35	24.61	223.81	2.34	4.63
38	6.11	16.09	146.34	2.28	4.50
39	5.06	13.32	121.12	2.40	4.70
40	3.56	9.36	85.11	2.81	5.55
41	4.48	11.79	107.23	3.31	6.54
42	7.19	18.93	172.12	3.77	7.46
43	4.38	11.52	104.79	3.34	6.59
44	4.59	12.09	109.90	2.79	5.51
45	4.74	12.47	113.40	4.74	9.37
46	4.56	11.99	109.06	3.17	6.27
47	4.52	11.89	108.12	2.26	4.46
48	3.41	8.97	81.61	2.13	4.20
49	3.98	10.47	95.19	2.38	4.71
50	3.45	9.07	82.48	2.05	4.05
51	4.90	12.90	117.31	2.88	5.68
52	3.95	10.39	94.45	3.28	6.48
53	3.47	9.14	83.07	3.30	6.52
54	5.70	14.50	136.40	4.31	8.52
55	3.46	9.10	82.70	2.69	5.31
56	8.70	22.90	208.20	6.24	12.33
57	5.63	14.81	134.71	6.58	13.01
58	2.77	7.28	66.20	3.14	6.20
59	5.73	15.08	137.12	3.07	6.07
60	6.34	16.68	151.66	3.18	6.29
61	4.64	12.22	111.12	3.98	7.87
62	4.83	12.71	115.58	2.95	5.83
63	6.20	16.31	148.33	3.16	6.24
64	3.32	8.75	79.55	2.38	4.70
65	3.33	8.77	79.76	2.45	4.83
66	2.36	6.21	56.45	2.12	4.18
67	3.04	8.00	72.74	2.32	4.59
68	4.02	10.58	96.25	3.78	7.46
69	5.37	14.13	128.45	6.46	12.77
70	6.06	15.96	145.11	3.95	7.81
71	4.14	10.90	99.10	4.11	8.13
72	4.03	10.60	96.42	2.83	5.59
73	4.75	12.50	113.69	4.57	9.04
74	3.85	10.14	92.20	2.33	4.61
75	2.95	7.78	70.70	2.32	4.59
76	4.49	11.82	107.44	3.22	6.37
77	3.56	9.36	85.15	3.46	6.84
78	2.44	6.41	58.31	2.86	5.66
79	2.07	5.46	49.64	3.08	6.07
80	1.99	5.25	47.74	2.26	4.47
81	2.68	7.05	64.07	2.39	4.73
82	4.01	10.55	95.90	2.49	4.93
83	1.58	4.15	37.72	1.99	3.93
84	2.16	5.69	51.77	2.47	4.88
85	2.17	5.72	52.03	2.57	5.07
86	2.48	6.54	59.42	4.13	8.17
87	2.54	6.69	60.79	2.40	4.75
88	1.78	4.68	42.59	2.44	4.83
89	2.53	6.67	60.60	2.35	4.64
90	2.95	7.77	70.68	2.27	4.48
91	2.38	6.27	57.00	2.44	4.82
92	3.07	8.08	73.46	2.44	4.44
93	2.40	6.33	57.55	2.34	4.62
94	3.32	8.73	79.40	2.02	3.99
95	3.82	10.06	91.50	2.55	5.04
96	3.62	9.53	86.66	2.07	4.08
97	4.44	11.69	106.33	2.39	4.72
98	3.79	9.97	90.67	2.96	5.85
99	2.59	6.83	62.08	2.36	4.66
100	2.29	6.04	54.93	2.47	4.89
101	2.99	7.87	71.59	2.54	5.03
102	4.19	11.04	100.36	3.91	7.74
103	6.86	18.07	164.35	3.75	7.41
104	4.10	10.79	98.11	2.60	5.14
105	5.93	15.62	142.07	4.00	7.92
106	3.01	7.94	72.18	4.21	8.31
107	6.85	18.04	164.05	2.85	5.64
108	4.63	12.02	110.96	2.94	5.81
109	3.53	9.29	84.52	2.75	5.37
110	4.32	11.37	103.41	2.90	5.73
111	7.09	18.66	169.67	3.63	7.17
112	4.52	11.91	108.33	2.84	5.61
113	3.96	10.43	94.88	4.62	9.14
114	2.82	7.42	67.43	2.88	5.68
115	2.87	7.56	68.70	2.25	4.45
116	4.24	11.17	101.57	1.75	3.45
117	4.60	12.12	110.17	2.08	4.12
118	3.00	7.89	71.76	4.09	8.08
119	2.46	6.48	58.95	3.43	6.78
120	2.61	6.88	62.56	3.34	6.60
121	2.81	7.38	67.15	4.09	8.09
122	13.67	36.00	327.34	2.96	5.85
123	9.26	24.37	221.60	2.64	5.22
124	5.44	14.33	130.32	2.43	4.80
125	8.28	21.81	198.31	4.50	8.89
126	8.89	23.41	212.92	5.77	11.40
127	7.26	19.10	173.69	3.19	6.31
128	11.19	29.46	267.90	9.35	18.48
129	13.86	36.49	331.82	8.96	17.71
130	6.86	18.05	164.15	4.96	9.80
131	6.31	16.60	150.99	4.55	8.98
132	5.65	14.86	135.17	3.03	6.00
133	6.59	17.34	157.70	2.84	5.62
134	5.60	14.74	134.02	3.82	7.55
135	6.96	18.31	166.54	2.59	5.13
136	4.87	12.81	116.50	2.06	4.07
137	6.85	18.03	163.97	1.97	3.90
138	6.24	16.43	149.41	2.08	4.10
139	3.37	8.88	80.74	2.24	4.42
140	5.41	14.24	129.47	2.89	5.72
141	6.28	16.52	150.25	2.90	5.73
142	4.68	12.33	112.13	2.89	5.72
143	4.07	10.71	97.43	2.84	5.61
144	4.39	11.56	105.16	2.50	4.95
145	4.13	10.86	98.76	2.42	4.79
146	4.49	11.81	107.41	3.33	6.59
147	7.17	18.87	171.59	3.54	7.00
148	13.01	34.24	311.39	7.75	15.32
149	6.14	16.15	146.89	4.42	8.74
150	5.82	15.31	139.24	5.75	11.37
151	4.86	12.79	116.31	4.18	8.26
152	10.35	27.25	247.79	7.88	15.58
153	10.82	28.47	258.93	9.25	18.29
154	7.19	18.93	172.12	9.08	17.94
155	6.81	17.92	162.98	5.83	11.52
156	6.71	17.67	160.66	9.35	18.48
157	6.44	16.95	154.16	7.09	14.02
158	8.96	23.59	214.55	7.02	13.87
159	7.72	20.34	184.92	6.08	12.02
160	4.67	12.29	111.73	3.71	7.33
161	3.57	9.41	85.57	2.85	5.63
162	4.26	11.21	101.89	1.91	3.78
163	3.59	9.45	85.97	2.15	4.25
164	4.46	11.75	106.81	2.31	4.56
165	4.12	10.86	98.73	2.30	4.54
166	2.77	7.30	66.35	2.39	4.72
167	3.57	9.41	85.55	2.53	4.99
168	4.87	12.83	116.64	2.24	4.43
169	4.80	12.63	114.87	2.82	5.57
170	4.68	12.31	111.94	4.18	8.27
171	4.25	11.20	101.84	3.60	7.12
172	5.84	15.37	139.74	3.65	7.21
173	3.48	9.17	83.41	4.23	8.36
174	6.16	16.22	147.51	3.16	6.25

Note: Column 1: region name. Column 2: integrated flux density of CO(1–0) emission. Column 3: molecular gas mass. Column 4: surface density of molecular hydrogen mass. Column 5: integrated flux of dust at 8 μm. Column 6: Star formation rate surface density.

**Table 3:** Integrated Flux Density, Neutral Atomic Gas Density and Mass*, f*_8_ μm dust and Star Formation Rate Surface Density.

Area	S_HI_ (Jy km s^−1^)	M_HI_ 10^−8^ (M_⊙_)	∑HI (M_⊙_ pc^−2^)	f_8_ μm (dust) (Jy)	∑SFR × 10^−2^ (M_⊙_ yr^−1^ kpc^−2^)
1	1.33	0.92	4.42	0.39	1.58
2	2.65	1.83	8.79	0.41	1.72
3	2.29	1.58	7.59	0.46	2.22
4	1.90	1.31	6.30	0.44	1.98
5	1.22	0.84	4.04	0.37	1.42
6	2.66	1.84	8.84	0.40	1.67
7	2.85	1.97	9.46	0.46	2.18
8	2.17	1.50	7.21	0.43	1.90
9	1.10	0.76	3.64	0.43	1.96
10	1.21	0.83	4.01	0.38	1.52
11	1.36	0.94	4.53	0.38	1.54
12	1.31	0.90	4.34	0.45	2.15
13	1.28	0.89	4.26	0.56	3.29
14	1.97	1.36	6.55	0.72	5.47
15	1.86	1.28	6.16	0.52	2.84
16	1.49	1.03	4.95	0.39	1.63
17	0.98	0.67	3.25	0.61	3.89
18	1.70	1.18	5.65	0.48	2.40
19	2.09	1.44	6.95	0.45	2.14
20	1.14	0.79	3.78	0.37	1.41
21	2.49	1.72	8.26	0.53	2.98
22	1.53	1.05	5.07	0.45	2.12
23	1.56	1.08	5.19	0.40	1.66
24	1.59	1.09	5.26	0.37	1.44

Note: Column 1: region name. Column 2: integrated flux density of HI emission line. Column 3: neutral atomic hydrogen mass. Column 4: surface density of neutral atomic hydrogen mass. Column 5: integrated flux of dust at 8 μm. Column 6: Star formation rate surface density.
